# Real-world treatment outcomes with halobetasol propionate 0.01%/tazarotene 0.045% lotion in patients with mild-to-moderate plaque psoriasis: A Canadian multicenter retrospective chart review

**DOI:** 10.1016/j.jdin.2022.05.001

**Published:** 2022-06-13

**Authors:** Ronald Vender, Irina Turchin, Perla Lansang, Vimal H. Prajapati, Mark Legault, Maxime Barakat, Jensen Yeung

**Affiliations:** a Department of Medicine, Division of Dermatology, McMaster University, Hamilton, Ontario, Canada; b Dermatrials Research Inc, Hamilton, Ontario, Canada; c Brunswick Dermatology Centre, Fredericton, New Brunswick, Canada; d Dalhousie University, Halifax, Nova Scotia, Canada; e Probity Medical Research, Waterloo, Ontario, Canada; f Division of Dermatology, Women’s College Hospital, Toronto, Ontario, Canada; g Division of Dermatology, Faculty of Medicine, University of Toronto, Toronto, Ontario, Canada; h Division of Dermatology, Department of Medicine, Sunnybrook Health Sciences Centre, University of Toronto, Toronto, Ontario, Canada; i The Hospital for Sick Children, Toronto, Ontario, Canada; j Division of Dermatology, Department of Medicine, University of Calgary, Calgary, Alberta, Canada; k Section of Community Pediatrics, Department of Pediatrics, University of Calgary, Calgary, Alberta, Canada; l Section of Pediatric Rheumatology, Department of Pediatrics, University of Calgary, Calgary, Alberta, Canada; m Dermatology Research Institute, Calgary, Alberta, Canada; n Skin Health & Wellness Centre, Calgary, Alberta, Canada; o Probity Medical Research, Calgary, Alberta, Canada; p Bausch Health Canada Inc, Laval, Quebec, Canada

**Keywords:** biologic, corticosteroid, fixed combination, plaque psoriasis, real-world, retinoid, retrospective chart review, topical

*To the Editor:* Halobetasol propionate 0.01%/tazarotene 0.045% lotion (HP/TAZ) was developed as an effective, safe, and tolerable topical therapy for plaque psoriasis. It has been investigated in moderate-to-severe plaque psoriasis^1^, ^2^, ^3^; however, real-world data on the use of topical fixed-combination therapies for plaque psoriasis, particularly milder disease courses, are limited.

In a pooled analysis of phase 3 studies involving patients with moderate-to-severe plaque psoriasis (baseline Investigator Global Assessment [IGA] score of 3/4 and affected body surface area [BSA] of 3%-12%),^2^^,^^3^ 40.6% of HP/TAZ-treated patients versus 9.9% of vehicle-treated patients achieved treatment success (IGA score of clear/almost clear [0/1] with ≥2-grade improvement from baseline) by week 8 (*P* < .001). HP/TAZ was also more effective than vehicle in reducing affected BSA (mean reduction: 37.6% vs 3.1%; *P* < .001).^1^

We conducted a Canadian multicenter retrospective chart review of 109 patients with mild-to-moderate plaque psoriasis defined as ≤7% BSA (as 5%-10% BSA is appropriate for defining moderate psoriasis).^4^ Patients with involvement of the face, intertriginous areas, and/or genitals were excluded. Eligible patients had initiated HP/TAZ (dosed in accordance with its product label) as monotherapy or as an adjunct to a stable dose of systemic therapy (ST) for ≥3 months. Patients using other topical medications were not excluded. The primary end point was the proportion of patients achieving treatment success (described above). The efficacy results reported herein are for week 8.

Most patients (95/109; 87.2%) maintained a stable dose of ST and/or HP/TAZ and completed 8 weeks of treatment (Table I). Overall, 43 of 95 (45.3%) patients achieved treatment success, which aligns with HP/TAZ in moderate-to-severe plaque psoriasis.^2^^,^^3^ A numerically higher proportion of patients receiving HP/TAZ monotherapy than HP/TAZ+ST achieved treatment success (33/58 [56.9%] vs 10/37 [27.0%]). IGA 0/1 was achieved by 63/95 (66.3%) of the overall population, with similar findings observed in the treatment subgroups (Fig 1). The absolute Psoriasis Area and Severity Index scores were lowered by 2.4 ± 1.6 (overall), 2.5 ± 1.7 (HP/TAZ monotherapy), and 2.1 ± 1.3 (HP/TAZ+ST), whereas the mean affected BSA was reduced by 47.8%, 56.7%, and 36.5%, respectively. For patients with scalp involvement (19/109 [17.4%])—the most common difficult-to-treat site^5^—13/17 (76.5%), 10/15 (66.7%), and 2/2 (100%), respectively, achieved treatment success, whereas the mean affected BSA reduction was consistently high across the treatment groups (Table I).

**Table I:** Efficacy, safety, and demographic and clinical characteristics of patients with mild-to-moderate plaque psoriasis treated with HP/TAZ

Variable	Overall *n* = 109	HP/TAZ M *n* = 67	HP/TAZ+ST *n* = 42∗†
Age, y; mean ± SD	49.7 ± 15.8	48.6 ± 17.4	51.4 ± 12.6
Sex, *n* (%):			
Female	51 (46.8)	29 (43.3)	22 (52.4)
Male	58 (53.2)	38 (56.7)	20 (47.6)
Sites of involvement, *n* (%):			
Lower limbs	77 (70.6)	52 (77.6)	25 (59.5)
Upper limbs	71 (65.1)	49 (73.1)	23 (54.8)
Trunk	35 (32.1)	21 (31.3)	15 (35.7)
Head and neck	28 (26.7)	17 (25.4)	11 (26.2)
Special site: scalp	27 (24.8)	16 (23.9)	11 (26.2)
Special site: palmoplantar	8 (7.3)	4 (6.0)	4 (9.5)
Prior topical therapies, *n* (%):			
None	5 (4.6)	5 (7.5)	0
1	33 (30.3)	15 (22.4)	18 (42.9)
2	27 (24.8)	19 (28.4)	8 (19.0)
3+	44 (40.4)	28 (41.8)	16 (38.1)
Prior systemic therapy, *n* (%):			
None	67 (61.5)	67 (100)	NA
Nonbiologic∗	6 (5.5)	NA	6 (14.3)
Biologic†	36 (33.0)	NA	36 (85.7)
*Overall population: all patients, regardless of the site of involvement*			
Baseline disease characteristics			
Affected BSA (%), mean ± SD	3.2 ± 1.7	3.5 ± 1.4	2.9 ± 1.9
PASI score, mean ± SD‡	4.1 ± 1.9	4.3 ± 1.9	3.0 ± 1.3
IGA score, *n* (%):			
1	10 (9.2)	1 (1.5)	9 (21.4)
2	31 (28.4)	12 (17.9)	19 (45.2)
3	68 (62.4)	54 (80.6)	14 (33.3)
Efficacy at week 8			
Affected BSA (%), mean ± SD§	1.8 ± 1.8	1.6 ± 1.4	2.1 ± 2.2
PASI score, mean ± SD‖	1.8 ± 1.6	1.9 ± 1.6	1.0 ± 0.7
IGA score, *n*/*N* (%):			
0	19/95 (20.0)	14/58 (24.1)	5/37 (13.5)
1	44/95 (46.3)	23/58 (39.7)	21/37 (56.8)
2	25/95 (26.3)	17/58 (29.3)	8/37 (21.6)
3	5/95 (5.3)	4/58 (6.9)	1/37 (2.7)
4	2/95 (2.1)	0	2/37 (5.4)
Treatment satisfaction at week 8, mean ± SD¶	6.6 ± 2.9	6.7 ± 2.9	6.4 ± 2.8
Adverse events leading to discontinuation up to week 8, *n* (%)	12 (11.1)	9 (13.4)	3 (7.1)
*Subpopulation: patients with scalp involvement*			
Baseline disease characteristics			
Affected BSA (%), mean ± SD#	1.2 ± 0.8	1.2 ± 0.9	1.3 ± 0.6
IGA score, *n*/*N* (%)#			
1	0/19 (0)	0/15 (0)	0/4 (0)
2	3/19 (15.8)	2/15 (13.3)	1/4 (25.0)
3	16/19 (84.2)	13/15 (86.7)	3/4 (75.0)
Efficacy at week 8			
Affected BSA (%), mean ± SD∗∗	0.4 ± 0.4	0.4 ± 0.4	0.5 ± 0.5
IGA score, *n/N* (%)∗∗			
0	8/17 (47.1)	7/15 (46.7)	1/2 (50.0)
1	5/17 (29.4)	4/15 (26.7)	1/2 (50.0)
2	4/17 (23.5)	4/15 (26.7)	0
*Safety in the overall population*			
Treatment-related AEs during 8 wks of treatment, *n* (%)			
Any AE	42 (38.5)	30 (44.8)	12 (28.6)
Irritation	35 (32.1)	28 (41.8)	7 (16.7)
Erythema	19 (17.4)	11 (16.4)	8 (19.0)
Burning	16 (14.7)	15 (22.4)	1 (2.4)
Pain	14 (12.8)	10 (14.9)	4 (19.5)
Pruritus	12 (11.0)	10 (14.9)	2 (4.8)
Atrophy	0	0	0
Hypopigmentation	0	0	0

*AE*, Adverse event; *BSA*, body surface area; *HP/TAZ*, halobetasol propionate 0.01%/tazarotene 0.045% lotion; *IGA*, Investigator Global Assessment; *M*, monotherapy; *NA*, not applicable; *PASI*, Psoriasis Area and Severity Index; *ST*, systemic therapy.

∗ Systemic nonbiologic therapies used by patients were acitretin (*n* = 3) and apremilast (*n* = 3).

† Systemic biologic therapies used by patients were adalimumab (*n* = 2), brodalumab (*n* = 1), certolizumab pegol (*n* = 1), etanercept (*n* = 2), guselkumab (*n* = 5), ixekizumab (*n* = 6), risankizumab (*n* = 5), secukinumab (*n* = 5), and ustekinumab (*n* = 9).

‡ Baseline PASI scores were available for 70 patients in the overall population, 60 patients in the HP/TAZ M group, and 10 patients in the HP/TAZ+ST group.

§ Week 8 affected BSA was available for 95 patients in the overall population, 58 patients in the HP/TAZ M group, and 37 patients in the HP/TAZ+ST group.

‖ Week 8 PASI score was available for 60 patients in the overall population, 52 patients in the HP/TAZ M group, and 8 patients in the HP/TAZ+ST group.

¶ Treatment satisfaction with HP/TAZ was captured on a 10-point scale where “1 = least satisfied” and “’10 = most satisfied.” Data were available for 98 patients in the overall population, 59 patients in the HP/TAZ M group, and 39 patients in the HP/TAZ+ST group.

# Baseline affected BSA and baseline IGA were available for 19 patients in the overall population, 15 patients in the HP/TAZ M group, and 4 patients in the HP/TAZ+ST group.

∗∗ Week 8 affected BSA and Week 8 IGA were available for 17 patients in the overall population, 15 patients in the HP/TAZ M group, and 2 patients in the HP/TAZ+ST group.

**Fig 1:**
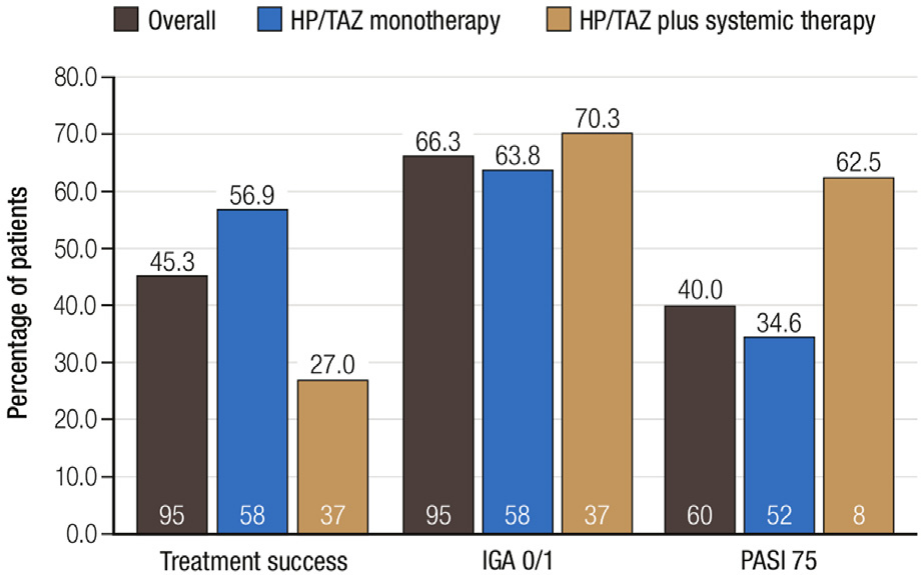
Plaque psoriasis. Proportion of patients achieving treatment success, IGA 0/1, or PASI 75 at week 8. Treatment success was defined as an IGA score of clear or almost clear (0/1) with a ≥2-grade improvement from baseline. Percentages were calculated based on the number of patients with IGA and PASI scores at baseline (corresponding number of patients completing week 8 assessments are shown at the base of each bar). *HP/TAZ*, Halobetasol propionate 0.01%/tazarotene 0.045% lotion; *IGA*, Investigator Global Assessment; *PASI*, Psoriasis Area and Severity Index; *PASI 75*, 75% improvement from baseline in Psoriasis Area and Severity Index score.

The most common adverse events occurring up to week 8 were irritation (32.1%), erythema (17.4%), and burning (14.7%). There were no cases of atrophy (rare in clinical studies^1^, ^2^, ^3^) or hypopigmentation. Discontinuation because of adverse events (11.1%) was somewhat higher than that reported in phase 3 studies (6.3%).^3^ Two patients receiving HP/TAZ+ST experienced worsening in the IGA score (1 case each of 1-grade and 2-grade worsening); the patient with 2-grade worsening subsequently discontinued the treatment. Overall, patients expressed moderate treatment satisfaction with HP/TAZ (mean rating: 6.6/10; Table I).

Despite the limitations commonly associated with retrospective chart reviews (eg, sampling size/bias, methodological constraints), collectively, this real-world analysis of HP/TAZ in Canadian patients with mild-to-moderate plaque psoriasis suggests that it is an effective, safe, and satisfactory treatment. This warrants large and prospective real-world studies in this patient population.

## Conflicts of interest

Dr Vender has served as a consultant, investigator, and/or received support or honoraria from AbbVie, Actelion, Amgen, Aralez, Arcutis, Bausch Health, Boehringer Ingelheim, BMS, Celgene, Cipher, Janssen, Galderma, GSK, Kabi-Care, Leo, Lilly, Merck, Novartis, Palladin, Pfizer, Sandoz, Sun Pharma, UCB, and Viatris-Mylan. Dr Turchin has served as consultant, speaker, or investigator for AbbVie, Amgen, Arcutis, Aristea, Bausch Health, BMS, Boehringer Ingelheim, Celgene, Eli Lilly, Galderma, Incyte, Janssen, Kiniksa, LeoPharma, Mallinckrodt, Novartis, Pfizer, Sanofi, Sun Pharma, and UCB. Dr Lansang has received honoraria and consulting fees from AbbVie, Amgen, Bausch, Celgene, Galderma, Janssen, Lilly, Novartis, Pfizer, Sanofi, UCB, and Valeant. Dr Prajapati has served as an investigator for AbbVie, Amgen, Arcutis, Arena, Asana, Bausch Health, Boehringer Ingelheim, Bristol Myers Squibb, Celgene, Concert, Dermavant, Dermira, Eli Lilly, Galderma, Incyte, Janssen, LEO Pharma, Nimbus Lakshmi, Novartis, Pfizer, Regeneron, Reistone, Sanofi Genzyme, UCB, and Valeant and has served as a consultant, advisor, and/or speaker for AbbVie, Actelion, Amgen, Aralez, Arcutis, Aspen, Bausch Health, Boehringer Ingelheim, Bristol Myers Squibb, Celgene, Cipher, Eli Lilly, Galderma, GlaxoSmithKline, Homeocan, Janssen, LEO Pharma, L'Oreal, Medexus, Novartis, Pediapharm, Pfizer, Sanofi Genzyme, Sun Pharma, Tribute, UCB, and Valeant. Dr Legault is an employee of Bausch Health Canada.

Dr Barakat is an employee of Bausch Health Canada. Dr Yeung has been a speaker, consultant, and investigator for AbbVie, Allergan, Amgen, Astellas, Bausch Health, Boehringer Ingelheim, Celgene, Centocor, Coherus, Dermira, Eli Lilly, Forward, Galderma, GSK, Janssen, Leo, Medimmune, Merck, Novartis, Pfizer, Regeneron, Roche, Sanofi Genzyme, Takeda, UCB, Valeant, and Xenon.
